# Protective Activity of Erythropoyetine in the Cognition of Patients with Parkinson’s Disease

**DOI:** 10.3390/bs8050051

**Published:** 2018-05-21

**Authors:** Ivonne Pedroso, Marité Garcia, Enrique Casabona, Lilia Morales, Maria Luisa Bringas, Leslie Pérez, Teresita Rodríguez, Ileana Sosa, Yordanka Ricardo, Arnoldo Padrón, Daniel Amaro

**Affiliations:** 1 International Center for Neurological Restoration, La Habana 11300, Cuba; merlincuba@nauta.cu (M.G.); enrique@neuro.ciren.cu (E.C.); lily@neuro.ciren.cu (L.M.); yordanka@neuro.ciren.cu (Y.R.); padron@neuro.ciren.cu (A.P.); 2 The Clinical Hospital of Chengdu Brain Science Institute, MOE Key Lab for Neuroinformation, University of Electronic Science and Technology of China, Chengdu 610054, China; 3 Center of Molecular Immunology, La Habana 11300, Cuba; leslie@cim.sld.cu (L.P.); teresita@cim.sld.cu (T.R.); daniel@cim.sld.cu (D.A.); 4 National Center for the Production of Laboratory Animal Breeding, La Habana 10800, Cuba; Iliana.sosa@cenpalab.cu

**Keywords:** cognitive, neuroprotection, Parkinson

## Abstract

**Introduction:** Treatment strategies in Parkinson’s disease (PD) can improve a patient’s quality of life but cannot stop the progression of PD. We are looking for different alternatives that modify the natural course of the disease and recent research has demonstrated the neuroprotective properties of erythropoietin. In Cuba, the Center for Molecular Immunology (CIM) is a cutting edge scientific center where the recombinant form (EPOrh) and recombinant human erythropoietin with low sialic acid (NeuroEPO) are produced. We performed two clinical trials to evaluate the safety and tolerability of these two drugs in PD patients. In this paper we want to show the positive results of the additional cognitive tests employed, as part of the comprehensive assessment. **Materials and method:** Two studies were conducted in PD patients from the outpatient clinic of CIREN, including *n* = 10 and *n* = 26 patients between 60 and 66 years of age, in stages 1 to 2 of the Hoehn and Yahr Scale. The first study employed recombinant human (rhEPO) and the second an intranasal formulation of neuroEPO. All patients were evaluated with a battery of neuropsychological scales composed to evaluate global cognitive functioning, executive function, and memory. **Results:** The general results in both studies showed a positive response to the cognitive functions in PD patients, who were undergoing pharmacological treatment with respect to the evaluation (*p* < 0.05) before the intervention. **Conclusions:** Erythropoietin has a discrete positive effect on the cognitive functions of patients with Parkinson’s disease, which could be interpreted as an effect of the neuroprotective properties of this molecules. To confirm the results another clinical trial phase III with neuroEPO is in progress, also designed to discard any influence of a placebo effect on cognition.

## 1. Introduction

Cognitive symptoms are not the feature that distinguishes Parkinson’s disease (PD) but they are very frequent in the natural evolution of this movement disorder [1] and are the cause of many of the disabilities [2]. These appear early [3], even before the onset of the parkinsonian syndrome and progress with it. They are characterized by difficulty in the formation of concepts, in temporal ordering and change of patterns, as well as alterations in attention, motor learning disorders, visuospatial disorders and memory dysfunction all related to the functions of the frontal lobe [4].

The alterations in the executive functions are the fundamental characteristic of the neuropsychological profile [5]. A significant number of patients develop dementia [5,6,7], which highly impacts their quality of life.

The evolution of cognitive symptoms is variable, from very slow in some cases to rapidly progressing towards dementia in others. The major risk factor is the time of progression and the severity of the motor symptoms, which correlates with the neuropathological stages [8]. Age is another important risk factor [9]. The combination with other non-motor symptoms such as depression and apathy make the patient more vulnerable to the development of dementia [10].

The neural substrate and the pathophysiology of cognitive impairment in PD are not fully known due to their complex nature and the multiple neurotransmission systems that are involved in them [5]. It is known that the pattern of onset of these symptoms is accompanied by alterations of the frontal-subcortical cortex, advancing the lesions towards the posterior cortical zones [7].

In the long term, these symptoms are the most disabling non-motor complications since they impair the mental functions in a permanent way, for up to now there is no way to control its clinical development. The response to the drugs used to treat these symptoms is very low and the progression is unstoppable.

The molecules that are studied for symptomatic treatment, and as neuroprotectors in PD, are mostly evaluated in terms of their action on motor symptoms, not in terms of their action on the neuropsychological sphere [11,12].

In the search for neuroprotective therapeutic alternatives, evidence has been found of the neuroprotective capacity of substances such as erythropoietin, a cytokine known as an important hematopoietic growth factor in tissue oxygenation [13]. It is a glycoprotein hormone that has 165 amino acids, weighs 30.4 kDa and is a member of the cytokine super families. Its location in the adult is the kidney while in the fetus its main production occurs in the liver [14,15].

In 1985 EPOrh was used for the first time in clinical treatments for patients in terminal stages of kidney diseases. In 1989, the US pharmaceutical and food industry approved its use. At present it is widely used in the treatment of anemia related to premature births, renal failure, cancer, chronic inflammatory diseases, and HIV infections [16,17,18].

Initially it was believed that its only function was to maintain tissue oxygenation at adequate levels; however, it has been shown to have other functions besides being an important hematopoietic factor [19].

The studies showed that EPO has other functions such as neuroprotection, which is performed by several mechanisms which are not all fully clarified. It reduces the toxicity of glutamate, induces the production of antiapoptotic factors, reduces inflammation, decreases the damage mediated by nitric acid, as well as has neurotrophic, antioxidant, and angiogenic action. It also promotes the formation of species reactive to oxygen, the activation of the protein kinase B (PKB) via the kinase-3 phosphoinositide, and the activation of the Janus Kinasa-2 (jak2) and the signaling factor of nuclear factor-kappaB (NF-kB) [19].

EPO has been identified as an important endogenous mediator of the tissue adaptive response to metabolic stress, capable of limiting the extent of tissue damage. In addition, it modifies both the neuronal electrical activity and the synthesis, transport, and release of neurotransmitters in dopaminergic and cholinergic populations, favoring the differentiation of dopaminergic neurons from their precursors under conditions of low oxygen availability in the central nervous system (CNS).

Studies have shown that EPO is capable of modifying the neuronal electrical activity as well as the synthesis, transport, and release of neurotransmitters in dopaminergic and cholinergic populations. Studies carried out in vitro on PC12 cells have demonstrated its ability to stimulate cell survival and increase dopamine release.

EPO is widely distributed in different regions of the body particularly susceptible to aging and atrophy such as the hippocampus, the substantia nigra, and the cerebral cortex. It has been demonstrated that its systemic administration has neuroprotective action in cultures of dopaminergic neurons in animal models MPTP and 6-OHDA of PD, subjected to hypoxia and ischemia induced by the deprivation of oxygen, glucose, glutamate, and excitotoxicity by nitric oxide; however, the striatal levels of catecholamines are maintained in normal limits with the response of a decrease in the rotational asymmetry of the mice [20,21,22]. In preclinical studies in PD as well as in other neurological [23,24,25] and psychiatric [26] diseases, EPO has also shown its capacity as a neuroprotector.

The EPOrh obtained in the Center of Molecular Immunology is registered and approved for use in humans in Cuba and other countries. The quality of the product is supported by its increasing international facilitates and its use in therapeutic clinical trials, with a subsequent generalization for use in an open population. In the production process of the EPOrh, isoforms with different sialic acid contents are obtained. When the weight of the EPO molecule is between 4 and 7 mmol/mL of protein, it is considered as having a low content of sialic acid (NeuroEPO). This molecule is similar to that produced in the brain of mammals but does not have an inducer effect in the synthesis of erythrocytes, maintaining its neuroprotective properties.

The name is due to its similarity with the EPO that is synthesized in the brain of mammals and is rapidly degraded by the liver due to its low content of sialic acid. Due to this it must be administered by a non-systemic route, such as intranasal [27,28]. Neuro-EPO is also obtained in the Molecular Immunological Center.

Supported by the knowledge of the characteristics of the molecule obtained through the investigations carried out, as well as the need for medicines and neuroprotective treatment strategies for PD and the economic feasibility of producing the molecule in our country, we carried out clinical studies with the use of this molecule.

We previously conducted a proof of concept clinical trial, administering EPOrh subcutaneously where the neuropsychological performance was measured as a secondary endpoint. Results showed an increased neuropsychological performance of patients after administration as compared to before administration results [29].

In a second investigation led by the author, this time using intranasal neuroEPO, with the main aim to show the safety and tolerability of the drug in PD patients, we took advantage of the neuropsychological evaluation to make a comparison with the first study.

The objective of the current study was to show the most significant results in relation to the neuropsychological scales in the current study performed with neuroEPO and make a comparison with those found previously in the study with EPOrh.

## 2. Materials and Methods

The first clinical trial was conducted at the International Center for Neurological Restoration CIREN www.ciren.cu and consisted of the administration of EPOrh subcutaneously at a dose of 60 IU/kg, once a week for five consecutive weeks. Ten patients with PD were included according to the Bank of London criteria in stages 1–3 of Hoehn and Yahr. Two patients were in stage 1, one in stage 1.5, one in stage II, five in stage 2.5 and one in stage 3. The age range was between 45 and 67 years with an average of 53 years. The distribution by sex was of 8 men and 2 women.

For the evaluation of cognitive symptoms, the Dementia Rating Scale Scale of Mattis (DRS) [30] was used. Evaluation was performed before starting the treatment, at week 1 of treatment, and 90 days after finishing it.

The statistical analysis was performed with the STATISTICA 6.0 package. The scores resulting from the evaluations were stored in databases.

Descriptive statistics such as the mean and the standard deviation were used for the statistical analysis to know the measurements of the central tendency of the variables and the test of the signs was employed to compare the differences between the scores obtained in the scales before and after the treatment with EPOrh.

All the patients signed an informed consent form as well as their relatives and the clinician conducting the trial.

The second study was conducted by two institutions: The International Center for Neurological Restoration and the Center for Molecular Immunology, who was the promoter. It was a phase I-II physician led clinical trial, in which 26 patients with PD who were classified as being in stages 1-2 according to the Hoehn and Yarh scale participated. The sample was randomly divided into two groups. Group A received neuroEPO and group B received placebo. The group A average age was 56.4 with a 7.8 standard deviation, females predominated at 53.4%, while patients in stage 2 made up 73.4% of the total. The evolution time had a mean of 5.4 years with a standard deviation of 3.2 and most of the patients were college graduates (46%).

In group B the mean age was 61.09 with a 6.6 standard deviation. The male sex prevailed, 72.7%, and stage 2 patients made up 80.8%. The evolution time average was 5.8 years with a standard deviation of 3.5 and most of the patients were college graduates (72.7%).

Despite the apparent differences between the clinical and demographic variables, the evaluation using the Mann-Whitney U and the chi square test did not show significant differences.

Randomization was performed by the CIM assigning N = 15 to group A and N = 11 to group B. The groups were respectively administered neuroEPO and placebo with identical organoleptic characteristics. The informed consent of all patients was obtained before the start of the trial.

The dose of neuroEPO was a vial with a dose of 1 mL/1mg administered intra-nasally for five consecutive weeks. The placebo group was administered 1 mL of an intranasal inert solution for the same period of time.

The assessment of cognitive symptoms was performed before starting the treatment, at week 1 of treatment and six months after its completion, with a wide-range battery of neuropsychological tests. These tests included the Mini mental Examination Test [31], DRS by Mattis, and The Frontal Assessment Battery (FAB) [32] to measure global cognitive functioning. The copy and reproduction of the Rey complex figure [33] was used to evaluate the visuoconstructive function and visual memory considering each one of the 18 units that compose it. The Verbal Fluency Test (D-KEFS) [34], which evaluated dorsolateral frontal lobe functions, consisted of verbal phonological fluency, verbal semantic fluency, and the capacity to alternate mental categories variables. The Word-Color Conflict Test (StroopTest) [35] gave us information about the state of selective and focused attention, the inhibition of responses, and the change of mental set. The Trail Making Test (TMT) [36] assessed the speed of visual location, attention, mental flexibility, working memory, and motor function. The Rey Verbal Auditory Learning Test [37] evaluated functions of the frontal lobe and had as variables working memory capacity, auditory-verbal learning ability, degree of retroactive interference, coding capacity, verbal memory storage, and ability to recognize auditory-verbal information. The working memory index of the WAIS III [38], a multidimensional battery, evaluates the state of the frontal lobe: integrated by subtests of sustained attention, working memory, and problem solving.

The IBM SPSS Statistics V 21 package was used for the statistical analysis of the data. We used tables of frequency analysis and descriptive statistics to analyze the demographic characteristics of the sample. The tables included means and percentages.

For the analysis of the results obtained by studying the differences between quantitative variables for paired and unpaired samples, the Wilcoxon and U of Mann–Whitney tests were used respectively. For the qualitative variables Chi square (X^2^) test was used. All values of *p* < 0.05 were considered significant.

## 3. Results

In the previous study performed with recombinant erythropoietin, administered subcutaneously, the results showed that the cognitive status of all patients measured only by the DRS Scale improved after treatment, their individual score being higher in the final evaluation with respect to the initial one (*z* = 2.84, *p*: 0.004).

The analysis of cognitive variables in the current study performed with intranasal neuroEPO had the following results:

**Phonological and Semantic Verbal Test (FAS)**:

Subtest the phonological verbal fluency: showed significant differences at week 1 after treatment (*z*: 2.2, *p*: 0.02,) in the group treated with neuroEPO, in comparison with the placebo group.

Subtest mental category change: significant differences were found in the group treated with neuroEPO (*z*: 2.13, *p* = 0.03) at six months after treatment.

**Dementia Rating Scale (DRS):**

Group treated with neuroEPO: showed significant differences between the pre-treatment evaluation, the week one (*p*: 0.01, *z*: 2.5), and six months after the end of treatment (*p*: 0.005, *z*: 2.8)

Placebo group: significant differences were found at week one after treatment (*p*: 0.01, *z*: 2.5) and at six months (*p*: 0.011, *z*: 2.5).

**Frontal Assessment Battery (FAB) Test of Litvan:**

Group treated with neuroEPO: significant differences in the evaluation made one week after the treatment (*p*: 0.009, *z*: 2.5) and six months later (*p*: 0.004, *z*: 2.8)

Placebo group: significant differences one week after treatment (*p*: 0.02, *z*: 2.3) and six months later (*p*: 0.009, *z*: 2.5)

**Rey complex figure**:

**Memory subtest:**

Group treated with neuroEPO: significant difference between pre-treatment, at one week (*p*: 0.0009, *z*: 3.2) and six months later evaluations (*p*: 0.001, *z*: 3.23) 

Placebo group: significant differences between the previous evaluation and one week (*p*: 0.01, *z*: 2.44) and six months after treatment (*p*: 0.007, *z*: 2.66).

**Copy subtest:**

Group treated with neuroEPO: had a significant difference in the evaluations conducted one week (*p*: 0.006, *z*: 2.7) and six months later after treatment (*p*: 0.007, *z*: 2.6).

Placebo group: also showed significant difference (*p*: 0.017, *z*: 2.3) but only six months after treatment.

The results with other tests, such as the Trail Making Test are not reported in this paper since significant differences were not found.

## 4. Discussion

In this study we focused on demonstrating some changes in the neuropsychological functions of a group of patients treated with two formulations of the molecule studied.

Our results are preliminary but the authors consider them to be relevant given the importance of cognitive symptoms in PD since they are one of the factors that most affects the quality of life of patients and caregivers, increasing social costs. They do not have a specific treatment and they evolve irreparably [39,40,41].

Within cognitive disorders in PD, executive alterations are the most important sign whether or not patients develop dementia and they tend to appear early in clinical evolution [42]. They appear accompanied by alterations in visuospatial abilities, spatial orientation, change of mental set, verbal fluidity mainly semantic, initiation, abstraction and generalization of thought, programming of behavior, and some modalities of memory and language [43,44].

Many investigations have shown that patients have symptoms of this type from the early stages of the disease [45].

The verbal fluency test measures the accomplishment of tasks of the set called executive functions that includes actions that require the use of underlying processes of access to the lexicon, and also the ability of cognitive organization, the ability to look for non-habitual words, focal and sustained attention as well as inhibition processes.

The executive functions are also responsible for the anticipation and setting of goals, the formation of plans and programs, the beginning of activities and mental operations, the temporal organization, sequencing, comparison, classification and categorization, the self-regulation of tasks, and the ability to carry them out efficiently [46,47].

These executive functions are processes that are directly linked to the coordinated functioning of the cortical and subcortical systems of the frontal lobes [48,49]. While evaluating them within our study we observed improvement in them, as measured by the verbal Fluency test, in the group exposed to neuroEPO. This was not so in the placebo group that was not exposed to the molecule, in which no significant differences were found in the evaluations after treatment.

In relation to the DRS, FAB of Litvan, and Rey complex figure tests we found that the results were positive for both groups, neuroEPO and placebo, which speaks in favor of the placebo effect.

The definition of the “placebo effect” [50] is well known in the field of research and clinical trials. It is used for the purpose of controlling the psychological effects of treatment.

In clinical research, a placebo is used intentionally to differentiate the pharmacological effects of the study drug from those unrelated to it. In this way it is possible to objectively separate the effects of the studied drug from others produced by the disease or by other factors.

Currently double-blind studies, in which one group of patients receives treatment with the drug under evaluation and the other receives only placebo, is the most adequate choice for the study of new drugs [51,52,53].

In our study this effect was also observed, for future clinical trials we plan to use tests that are not very susceptible to this effect.

The authors found promising results in the sphere of neuropsychology in PD while investigating neuroprotection. The drug, even administered at low doses because the studies conducted were safety studies, had interesting results, which suggests a possible effect of erythropoietin on the cognitive sphere, as reported already in preclinical studies.

Because this result is very preliminary it is necessary to conduct new studies with an adequate design to demonstrate the possible benefit in cognitive functions. To this aim a Phase II-III clinical trial is being conducted in which we intend to assess whether or not there is a positive impact of the molecule on the symptoms of the disease and its capacity for neuroprotection, given the need for this type of therapy in PD and the necessity to advance in the understanding of the cellular mechanisms of neurodegeneration.

Our studies have several limitations, one of them was that the first study did not have a control group. The other limitation is since both studies were clinical safety trials, small doses of the drug were administered.

However, in spite the fact that the positive impact of EPO on cognition in preclinical and clinical studies has been established in the literature, it has also established that this impact is dose dependent [54]. These findings highlight the need to continue studying the effect of erythropoietin on cognitive functions in PD using higher doses.

On the other hand, the results obtained were based on the total scores of the scales employed, however, we intend in the near future to study the cognitive latent variables related with the drug, which can be revealed using item-response theory, in terms of evaluating the discriminative power of individual items instead of the global scores of the neuropsychological scales.

## 5. Conclusions

In conclusion, the authors suggest that the beneficial effect in patients undergoing treatment with both EPOrh and neuroEPO could be an effect of the molecules, but since the placebo effect is present, further studies will be necessary to demonstrate the neuropsychological benefits.
